# A Coupled Electro-Mechanical Homogenization-Based Model for PVDF-Based Piezo-Composites Considering α → β Phase Transition and Interfacial Damage

**DOI:** 10.3390/polym15142994

**Published:** 2023-07-10

**Authors:** Fateh Enouar Mamache, Amar Mesbah, Fahmi Zaïri, Iurii Vozniak

**Affiliations:** 1 University of Sciences and Technology Houari Boumediene, Laboratory of Advanced Mechanics, Algiers 16111, Algeria; 2 Univ. Lille, IMT Nord Europe, JUNIA, Univ. Artois, ULR 4515—LGCgE, Laboratoire de Génie Civil et géo-Environnement, F-59000 Lille, France; 3 Centre of Molecular and Macromolecular Studies, Polish Academy of Sciences, Sienkiewicza Str., 112, 90363 Lodz, Poland; wozniak@cbmm.lodz.pl

**Keywords:** piezoelectric composites, micromechanical model, α → β phase transition, damage

## Abstract

Polyvinylidene fluoride or polyvinylidene difluoride (PVDF) is a piezoelectric semi-crystalline polymer whose electro-mechanical properties may be modulated via strain-induced α → β phase transition and the incorporation of polarized inorganic particles. The present work focuses on the constitutive representation of PVDF-based piezo-composites developed within the continuum-based micromechanical framework and considering the combined effects of particle reinforcement, α → β phase transition, and debonding along the interface between the PVDF matrix and the particles under increasing deformation. The micromechanics-based model is applied to available experimental data of PVDF filled with various concentrations of barium titanate (BaTiO_3_) particles. After its identification and predictability verification, the model is used to provide a better understanding of the separate and synergistic effects of BaTiO_3_ particle reinforcement and the micromechanical deformation processes on the electro-mechanical properties of PVDF-based piezo-composites.

## 1. Introduction

Multifunctional materials have become centerpieces of our modern society. They combine the performance objectives of several components into a single material system. As a relevant example, piezoelectric materials couple electrical and mechanical fields and have the ability of transferring from mechanical energy to electrical energy and vice versa. Their electroactive abilities can be exploited in various engineering applications, such as sensors/actuators and energy generation/storage [1,2]. This work is dedicated to PVDF (polyvinylidene fluoride or polyvinylidene difluoride), which is a semi-crystalline polymer with dielectric and electroactive abilities, including piezoelectric, pyroelectric and ferroelectric effects [3,4]. PVDF electroactivity is closely related to its microstructure, and the material is employed as is or reinforced by particles. Establishment of the relationship between structure (crystallinity and particle reinforcement) and mechanical/piezoelectric properties of PVDF is fundamental for a reliable design of such material systems. PVDF crystallinity is manifested by four crystalline forms, namely, α, β, γ and δ [5]. When cooling from the melt, the commonly obtained crystalline form in PVDF is in the α-phase, but it is non-polar, non-piezoelectric, and non-pyroelectric [4]. Regarding electroactive abilities, there is great interest in involving the polarized β-phase in PVDF [6], which exhibits strong ferroelectric and piezoelectric properties. These may be obtained via different experimental techniques [7], one being the transition from the α-phase to the β-phase crystalline structure in the course of a mechanical stretching under suitable loading conditions in terms of temperature and strain rate [8,9,10]. The piezoelectric properties will depend thus on the β-phase content and its characteristics [11,12]. Moreover, the insertion of inorganic particles is a common practice to reinforce PVDF [13,14,15], and, when polarized, to combine the mobility of the dipoles present in PVDF. Optimization of the composite formulation in terms of particle features and processing parameters is often necessary to achieve the desired electro-mechanical properties. The specific effect of the inserted particles can vary depending on interfacial interactions with the PVDF matrix. Weak interfacial interactions always weaken the composite mechanical properties, but also introduce higher electrical resistance at the interfaces, leading to a decrease in the overall polarization. Undoubtedly, the identification of the separate and synergistic effects of the β-phase features (and the associated loading conditions for the α → β phase transition) and of the particle features is difficult to assess experimentally.

Establishment of a quantitative relationship between mesostructural properties and electro-mechanical properties of the semi-crystalline PVDF system is of prime importance for advanced engineering applications. Accurate and flexible predictive tools allowing tailoring and optimizing this relationship become thus precious in terms of time and cost saving. It is now recognized that continuum-based modeling is helpful, provided that the constitutive representation of the material system is as physically realistic as possible. In a very recent work, Yan et al. [16] developed a micro-macro constitutive model for the large-strain plastic deformation description by regarding at the mesoscopic scale the semi-crystalline PVDF medium (being transformed) as an aggregate of three-phase (α-β-amorphous) layered composite inclusions, but without considering the presence of particles. It is nonetheless important to consider the specific effect of particle reinforcement and interfacial interactions in the modeling approach in order to offer an accurate predictive tool to understand and predict the microstructure–property relationship of PVDF-based piezo-composites, taking into account the α → β phase transition. Considering the fact that micro-macro modeling based on Eshelby-type solutions provides more detailed insights into the local interactions, the present study will be settled within this theoretical framework.

Over the years, micro-macro homogenization-based models through Eshelby-type inclusion problems have been proposed to predict the effective behavior of piezoelectric composites. The models developed within the Eshelby-type inclusion approach especially allow an explicit consideration of the local interactions between the inclusions and the surrounding matrix. In the case of piezoelectric media, extensions of Eshelby elastic tensors for ellipsoidal/spheroidal inclusions were proposed [17,18,19]. Wang [20] developed an approach for the coupled electroelastic field in piezoelectric ellipsoidal inclusions embedded in an infinite matrix. Azar’s research team [21,22,23] used the Mori–Tanaka model to investigate some of the effective properties of multiphase piezoelectric material constants. Odegard [24] and later Wang et al. [25] proposed models based on the Mori–Tanaka and self-consistent approaches to predict the electroelastic properties of piezoelectric composites. The Mori–Tanaka approach was also extended to multiphase piezoelectric composites by Malakooti and Sodano [26]. The electroelastic behavior of piezoelectric fiber-reinforced composites was also constitutively modeled by Eynbeygi and Aghdam [27]. Using this modeling approach, Eynbeygi and Aghdam [28] simulated the thermo-electroelastic behavior of transversely polarized piezoelectric fibrous composites. Mahmoodi et al. [29] proposed a constitutive model trough a hierarchical approach to relate the structural parameters to the electroelastic properties of laminated carbon nanotube/PVDF systems. To the best of our knowledge, no Eshelby-type micromechanical model has considered strain-induced structure changes, such as α → β phase transition, and their effect on the electroelastic properties.

The main objective of this paper is to propose a coupled electro-mechanical micromechanical model for PVDF able to consider the effective role of polarized inorganic particles in the composite response in relation to α → β phase transition and interfacial debonding caused by increasing strain. After its identification and predictability verification on available experimental data of PVDF particulate composites, the model is used to clarify the connection between particle reinforcement, micromechanical deformation processes, and electroelastic properties.

The paper is organized as follows. Section 2 is devoted to the description of the micromechanics-based constitutive equations. Section 3 presents the comparisons of the model outputs with existing experiments. Concluding remarks are finally given in Section 4.

## 2. Constitutive Equations

This section describes the constitutive equations to predict the effective properties of heterogeneous piezoelectric composites based upon the continuum-based micromechanical framework. They are treated as an Eshelby-type inclusion problem in which the reinforced semi-crystalline material system is regarded as a continuous amorphous matrix (phase am) in which discrete ellipsoidal inclusions are randomly dispersed and oriented, namely, the (α and β) crystalline phases and the particles assumed initially to be perfectly bonded at interfaces. Such a composite-type representation of the semi-crystalline structure was previously employed to predict different behaviors in the context of elasticity [30,31,32], plasticity [33] and plasticity with strain-induced phase transformation [34]. The progressive interfacial debonding and α → β phase transition are the two prevalent deformation mechanisms considered in the present work and introduced into the micromechanical framework. In addition to intact particles (phase $P_{1}$), partially and fully damaged particles (phases $P_{2}$ and $P_{3}$) may likely develop when the interfacial debonding occurs.

The following notation is used throughout the text. Tensors are denoted by boldface letters, while scalars and individual components of vectors and tensors are denoted by normal italicized letters. The superposed dot designates the time derivative. The superscript *T* indicates the transpose quantity.

### 2.1. Piezoelectric Multiphase Composites

The modified Hooke’s law constitutively relates the macroscopic stress ${\bar{\sigma }}_{ij}$ to the macroscopic elastic strain ${\bar{\varepsilon }}_{ij}^{e}$ and the electric field ${\bar{E}}_{n}$:

(1) $${\bar{\sigma }}_{ij}={\bar{C}}_{ijmn}{\bar{\varepsilon }}_{mn}^{e}-{\bar{e}}_{nij}{\bar{E}}_{n}$$

where ${\bar{C}}_{ijmn}$ is the macroscopic elastic stiffness tensor and ${\bar{e}}_{nij}$ is the piezoelectric tensor.

The electric displacement ${\bar{D}}_{i}$ is given by:

(2) $${\bar{D}}_{i}={\bar{e}}_{imn}{\bar{\varepsilon }}_{n}^{e}\;+{\bar{\kappa }}_{in}{\bar{E}}_{n}$$

where ${\bar{\kappa }}_{in}$ is the relative permittivity matrix and ${\bar{E}}_{n}$ is the electric field vector.

For convenience in the micromechanical developments for piezoelectric problems, a single matrix form can be employed to combine electrical and mechanical properties via $\bar{\Sigma }=\bar{E}\bar{Z}$, written, by means of the engineering matrix form, as:

(3) $$\left[\begin{array}{c} {\bar{\sigma }}_{11}^{}\\ {\bar{\sigma }}_{22}^{}\\ {\bar{\sigma }}_{33}^{}\\ {\bar{\sigma }}_{23}^{}\\ {\bar{\sigma }}_{13}^{}\\ {\bar{\sigma }}_{12}^{}\\ {\bar{D}}_{1}\\ {\bar{D}}_{2}\\ {\bar{D}}_{3} \end{array}\right]=\left[\begin{array}{ccccccccc} {\bar{C}}_{11} & {\bar{C}}_{12} & {\bar{C}}_{13} & 0 & 0 & 0 & 0 & 0 & -{\bar{e}}_{31}\\ {\bar{C}}_{12} & {\bar{C}}_{22} & {\bar{C}}_{23} & 0 & 0 & 0 & 0 & 0 & -{\bar{e}}_{32}\\ {\bar{C}}_{13} & {\bar{C}}_{23} & {\bar{C}}_{33} & 0 & 0 & 0 & 0 & 0 & -{\bar{e}}_{33}\\ 0 & 0 & 0 & {\bar{C}}_{44} & 0 & 0 & 0 & -{\bar{e}}_{15} & 0\\ 0 & 0 & 0 & 0 & {\bar{C}}_{55} & 0 & -{\bar{e}}_{15} & 0 & 0\\ 0 & 0 & 0 & 0 & 0 & {\bar{C}}_{66} & 0 & 0 & 0\\ 0 & 0 & 0 & 0 & {\bar{e}}_{15} & 0 & {\bar{\kappa }}_{1} & 0 & 0\\ 0 & 0 & 0 & {\bar{e}}_{15} & 0 & 0 & 0 & {\bar{\kappa }}_{2} & 0\\ {\bar{e}}_{31} & {\bar{e}}_{32} & {\bar{e}}_{33} & 0 & 0 & 0 & 0 & 0 & {\bar{\kappa }}_{3} \end{array}\right]\left[\begin{array}{c} {\bar{\varepsilon }}_{11}^{e}\\ {\bar{\varepsilon }}_{22}^{e}\\ {\bar{\varepsilon }}_{33}^{e}\\ {\bar{\gamma }}_{23}^{e}\\ {\bar{\gamma }}_{13}^{e}\\ {\bar{\gamma }}_{12}^{e}\\ {\bar{E}}_{1}\\ {\bar{E}}_{2}\\ {\bar{E}}_{3} \end{array}\right]$$

where $\bar{\Sigma }$ is a tensor including stress and electric displacement fields, $\bar{Z}$ is a tensor including elastic strain and electric fields, and $\bar{E}$ is the electroelastic property matrix:

(4) $$\bar{E}=\left[\begin{array}{cc} {\bar{C}}_{6\times 6} & -{\bar{e}}_{6\times 3}^{T}\\ {\bar{e}}_{3\times 6} & {\bar{\kappa }}_{3\times 3} \end{array}\right]$$

By adopting the micromechanical homogenization solution of Ju and Sun [35], the macroscopic electroelastic property tensor $\bar{E}$ of the multiphase composite can be given by:

(5) $$\bar{E}=E_{am}\cdot \left\{I-\left(Y_{\alpha }+Y_{\beta }+\sum_{i=1}^{3}Y_{P_{i}}\right)\cdot {\left[\left(S_{\alpha }\cdot Y_{\alpha }+S_{\beta }\cdot Y_{\beta }+\sum_{i=1}^{3}S_{P_{i}}\cdot Y_{P_{i}}\right)+I\right]}^{-1}\right\}$$

where I is the identity tensor, $S_{\alpha }$, $S_{\beta }$ and $S_{P_{i}}$ are the electroelastic Eshelby tensors defined by the expressions of Mikata [18], and $Y_{\alpha }$, $Y_{\beta }$ and $Y_{P_{i}}$ are three fourth-order tensors expressed as:

(6) $$Y_{\alpha }=-\phi_{\alpha }{\left(S_{\alpha }+A_{\alpha }\right)}^{-1},\;Y_{\beta }=-\phi_{\beta }{\left(S_{\beta }+A_{\beta }\right)}^{-1}\;\mathrm{and}\;Y_{P_{i}}=-\phi_{P_{i}}{\left(S_{P_{i}}+A_{P_{i}}\right)}^{-1}$$

where $\phi_{\alpha }$, $\phi_{\beta }$ and $\phi_{P_{i}}$ (i=1,2,3) are the volume fractions and $A_{\alpha }$, $A_{\beta }$ and $A_{P_{i}}$ are the electroelastic mismatch tensors, respectively, defined as:

(7) $$A_{\alpha }={\left(E_{\alpha }-E_{am}\right)}^{-1}\cdot E_{am},\;A_{\beta }={\left(E_{\beta }-E_{am}\right)}^{-1}\cdot E_{am}\;\mathrm{and}\;A_{P_{i}}={\left(E_{P_{i}}-E_{am}\right)}^{-1}\cdot E_{am}$$

which are written as a function of the electroelastic property matrices:

(8) $$E_{\alpha }=\left[\begin{array}{cc} C_{\alpha } & 0\\ 0 & \kappa_{\alpha } \end{array}\right],\;E_{\beta }=\left[\begin{array}{cc} C_{\beta } & -e_{\beta }^{T}\\ e_{\beta } & \kappa_{\beta } \end{array}\right],\;E_{P_{i}}=\left[\begin{array}{cc} C_{P_{i}} & -e_{P_{i}}^{T}\\ e_{P_{i}} & \kappa_{P_{i}} \end{array}\right]\;\mathrm{and}\;E_{am}=\left[\begin{array}{cc} C_{am} & 0\\ 0 & \kappa_{am} \end{array}\right]$$

The inherent coupling between electric and mechanical fields being induced by the β-PVDF and the inserted particles, the only ones to be polarized, the extra-diagonal elements for the α-PVDF, and the amorphous phase are null.

### 2.2. Micromechanical Deformation Processes

#### 2.2.1. Strain-Induced α → β Phase Transition

In order to relate the macroscopic behavior to the microstructure evolution, the progressive α → β phase transition under applied macroscopic deformation may be included in the micromechanical model using the following relations:

(9) $$\phi_{\alpha }=\phi_{\alpha \_0}-\phi_{\beta }\;\mathrm{and}\;\phi_{\beta }=\phi_{\beta }{}_{\_\infty }\chi$$

in which the volume fraction of α-phase $\phi_{\alpha }$ follows a decreasing function under increasing deformation while the mechanism of α → β phase transition occurs. The term $\phi_{\alpha \_0}$ is the initial volume fraction of α-phase, $\phi_{\beta }{}_{\_\infty }$ is the maximum volume fraction of β-phase reached upon the mechanical loading (equal to $\phi_{\alpha \_0}$ for a full transformation) and χ is the total degree of transformation expressed using an Avrami-type expression as follows [36]:

(10) $$\dot{\chi }=\frac{\dot{\varepsilon }}{{\dot{\varepsilon }}_{ref}}\alpha_{av}K_{av}{\left(-\ln\left(1-\chi \right)\right)}^{\frac{\alpha_{av}-1}{\alpha_{av}}}\left(1-\chi \right)$$

in which $\dot{\varepsilon }$ is the maximum applied strain rate, ${\dot{\varepsilon }}_{ref}$ is the reference strain rate, $\alpha_{av}$ is the Avrami exponent and $K_{av}$ is the phase transformation rate given in the case of PVDF by the empirical form [16]:

(11) $$K_{av}=0.75\times 10^{-3}{\left(\frac{4\pi Nu}{3\phi_{\beta \_\infty }}\right)}^{1/3}\exp\left(-{\left(\frac{\theta -206}{47.33}\right)}^{2}\right)$$

in which θ is the absolute temperature and Nu represents the number density of nuclei.

The latter formulation was initially proposed for spherulitic growth in thermally induced crystallization. Although it is not an optimized choice for all kinetics of newly formed crystals due to differences in morphology and in size [37], it was already used in the context of strain-induced phase transformation [16,38,39,40]. It was slightly adjusted to represent the α → β phase transition in neat PVDF by Yan et al. [16] using the experimental large-strain stress–strain data of Defebvin [9].

#### 2.2.2. Interfacial Debonding

The loading conditions affect the α → β phase transition, but also the elementary plastic deformation mechanisms. Several experiments reported in the literature evidenced the occurrence of a cavitation phenomenon into the PVDF amorphous phase [10,41]. The former one plays an important role for neat semi-crystalline PVDF at straining temperatures lower than 60 °C and can be ignored for higher temperatures. In the case of filled PVDF, debonding along the interface between the PVDF matrix and the particles can occur. The progressive degradation of particles acts as a weakening factor of the electro-mechanical properties. In the model, three populations of particles are considered. The first one, termed $P_{1}$, represents the intact particles, while the two others, termed $P_{2}$ and $P_{3}$, denote, respectively, the partially and fully debonded particles from the PVDF matrix. The progressive evolution of volume fractions of particles is described in accordance with Weibull statistical functions introduced in a sequential manner to reproduce the transfer from partial debonding to full debonding under increasing macroscopic deformation:

(12) $$\begin{array}{l} \phi_{P_{2}+P_{3}}=\phi_{P_{2}}+\phi_{P_{3}}=\phi_{P}\left\{1-\exp{\left[-\left(\frac{\left\|\bar{\varepsilon }\right\|}{s}\right)\right]}^{m}\right\}\\ \phi_{P_{3}}=\phi_{P_{2}+P_{3}}\left\{1-\exp{\left[-\left(\frac{\left\|\bar{\varepsilon }\right\|}{s}\right)\right]}^{m}\right\}\\ \phi_{P_{1}}=\phi_{P}-\phi_{P_{2}+P_{3}}\\ \phi_{P_{2}}=\phi_{P}-\phi_{P_{1}}-\phi_{P_{3}} \end{array}$$

in which $\phi_{P}$ is the original volume fraction of bonded particles, m and s are the damage parameters, and $\left\|\bar{\varepsilon }\right\|$ is the Frobenius norm of the macro-strain tensor.

To consider the partial local stress transfer in the partially debonded particles, they are replaced by equivalent perfectly bonded particles with orthotropic properties and allowing vanishing of the local normal and shear stresses in the debonded direction [42,43]. The loss of local stress transfer in any direction in the fully debonded particles is introduced by the vanishing of the corresponding stiffness. Once the interfacial debonding occurs, it is assumed that the damaged particle does not act in the polarization anymore.

### 2.3. Piezoelectric Activity

In α → β phase transition, the piezoelectric coefficient $d_{3i\_PVDF}$ (resp. the dielectric constant $\kappa_{3i\_PVDF}$) varies upon straining from its initial value $d_{3i\_PVDF}^{0}$ (resp. $\kappa_{3i\_PVDF}^{0}$) to its final value $d_{3i\_PVDF}^{\infty }$ (resp. $\kappa_{3i\_PVDF}^{\infty }$). The expression provided by Wang et al. [25] is employed to estimate the evolution of the PVDF piezoelectric coefficient $d_{3i\_PVDF}$ as a function of the β-phase volume fraction $\phi_{\beta }$:

(13) $$d_{3i\_PVDF}=\left[\left(\frac{15\phi_{\beta }}{\left(2+3\phi_{\beta }\right)\left(1-\phi_{\beta }\right)}\frac{\kappa_{ii\_PVDF}^{0}}{\kappa_{ii\_PVDF}^{\infty }}d_{3i\_PVDF}^{\infty }\right)+d_{3i\_PVDF}^{0}\right]$$

in which the dielectric constants $\kappa_{ii\_PVDF}^{0}$ and $\kappa_{ii\_PVDF}^{\infty }$ are taken to be equal to those of α crystals and β crystals, respectively.

The macroscopic piezoelectric coefficient ${\bar{d}}_{3i}$ is in turn given as a function of the particle volume fraction $\phi_{P}$:

(14) $${\bar{d}}_{3i}=\left[\left(\frac{15\phi_{P}}{\left(2+3\phi_{P}\right)\left(1-\phi_{P}\right)}\frac{\kappa_{ii\_PVDF}^{\infty }}{\kappa_{ii\_P}}d_{3i\_P}\right)+d_{3i\_PVDF}\right]$$

The macroscopic dielectric constant ${\bar{\kappa }}_{ii}$ is further estimated considering full α → β transformation as follows [25]:

(15) $${\bar{\kappa }}_{ii}=\left(\frac{1+2\phi_{P}}{1-\phi_{P}}\kappa_{ii\_PVDF}^{\infty }\right)$$

The model inputs include the components of the electroelastic property matrices for the different phases and the phase degrees, including the constants related to phase transition and damage.

## 3. Model Application

The micromechanics-based model was entirely coded in Matlab software. Its capabilities to capture the relation between structure and electro-mechanical properties of PVDF-based composites are examined by comparing model simulation outputs to available experimental data taken from the work of Defebvin [9] (see also [44]). The material is PVDF filled with various concentrations of barium titanate (BaTiO_3_) particles varying from 0 to 30 wt%. Defebvin [9] showed that the BaTiO_3_ particles are individually dispersed into the PVDF matrix for the whole range of concentrations kept here. The particle volume fraction $\phi_{P}$ can be expressed as:

(16) $$\phi_{P}={\left(1+\left(\frac{1}{W_{P}}-1\right)\frac{\rho_{P}}{\rho_{PVDF}}\right)}^{-1}$$

where $W_{P}$ is the particle weight fraction, $\rho_{P}$ = 5.82 g/cm^3^ is the BaTiO_3_ density [45] and $\rho_{PVDF}=\phi_{a}\rho_{a}+\phi_{\alpha }\rho_{\alpha }+\phi_{\beta }\rho_{\beta }$ is the PVDF density, with the densities of amorphous $\rho_{a}$, pure α-PVDF $\rho_{\alpha }$ and pure β-PVDF $\rho_{\beta }$ equal to 1.68, 1.92 and 1.97 g/cm^3^, respectively [16,46,47]. The presence of BaTiO_3_ particles does not induce a change in the crystalline structure of PVDF, even at high amounts [9,44]. The PVDF matrix is thus initially crystallized in the non-polar crystalline α-phase with an initial α-phase content $\phi_{\alpha \_0}$ of 70%. The electroactive β-phase can be obtained by straining, provided that the composite integrity is preserved.

### 3.1. Model Constants

#### 3.1.1. Piezoelastic Constants

Table 1 lists the elastic components of the crystalline phases $C_{\alpha \_ij}$ and $C_{\beta \_ij}$, taken from the density-functional theory work of Pei and Zeng [48]. Rather close values were found with the molecular dynamics simulations of Yan et al. [16]. Those of the intact particles $C_{P_{1}}{}_{\_ij}$ are also taken from the literature [21] and listed in Table 1.

The piezoelectric constants of the BaTiO_3_ particles are taken from the paper of Fakri et al. [21], namely, $\left\{e_{31\_P},e_{33\_P},e_{15\_P}\right\}$ = {−4.4 C/m^2^, 18.6 C/m^2^, 11.6 C/m^2^} and $\left\{\kappa_{11\_P}/\kappa_{0},\kappa_{33\_P}/\kappa_{0}\right\}$ = {1665.5, 1423.7}, $\kappa_{0}$ = 8.85 × 10^−12^ C^2^/Nm^2^ being the permittivity of free space. The dielectric constants of α and β crystals are taken from the works of Venkatragavaraj et al. [49] and Defebvin [9]: respectively, $\kappa_{33\_PVDF}^{0}/\kappa_{0}$ = 12 and $\kappa_{33\_PVDF}^{\infty }/\kappa_{0}$ = 24. In addition to the spherical BaTiO_3_ particles, the α and β crystalline forms are two reinforcement elements of the amorphous matrix, for which identical shape factors are imposed. The amorphous phase is assumed to be isotropic and its elastic constants are assumed to be independent of both the relative extent of crystalline forms and the particle concentration. Since the region of interest is the rubbery one, the amorphous Poisson’s ratio $\nu_{am}$ is taken constant. The variation in amorphous Young’s modulus $E_{am}$ with the temperature θ is described using the following expression [50]:

(17) $$E_{am}\left(\theta \right)=\frac{1}{2}\left(E_{g}+E_{r}\right)-\frac{1}{2}\left(E_{g}-E_{r}\right)\tanh\left(\frac{5}{\Delta \theta }\left(\theta -\theta_{g}\right)\right)+X_{g}\left(\theta -\theta_{g}\right)$$

where $E_{g}$ and $E_{r}$ are the amorphous moduli in the glassy and rubbery regions, respectively, Δθ is the interval of the temperature range across which the glass transition $\theta_{g}$ occurs and $X_{g}$ is the slope outside the glass transition region.

**Figure 1:**
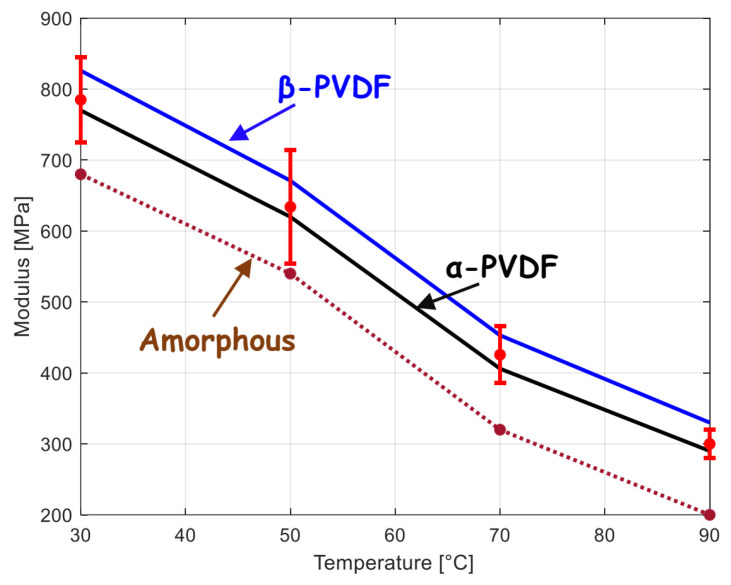
Elastic stiffness as a function of temperature for neat amorphous PVDF and neat crystallized PVDF containing only α or β crystals (lines: model, symbols: experimental data from [51]).

The different constants, together with the crystal shape factor, were calibrated using the overall stiffness data extracted from the Laiarinandrasana et al. [51] paper on neat PVDF crystallized in the α form. Figure 1 shows quite a good reproduction of the experimental stiffness by the theoretical fit with temperature variation. The identified values are listed in Table 2. The structure–stiffness relationship is also pointed out in Figure 1 by considering a purely amorphous PVDF and a PVDF crystallized in the β form with the same crystal amount as that used for the theoretical fit. The two theoretical curves constitute, respectively, a lower bound and an upper bound of the modulus variation with temperature. It is worth noting that the inverse method used here to obtain the amorphous elastic stiffness using the experimental data of Laiarinandrasana et al. [51] leads to a relatively higher value than that obtained by Yan et al. [16] using molecular dynamics simulations of a purely amorphous PVDF system.

#### 3.1.2. Phase Transition Constants

The progressive micro-mechanism of α → β phase transition is highly dependent on the loading conditions, such as the temperature that induces modifications on the overall material behavior [9,10]. Defebvin [9] reported full α → β phase transition (i.e., $\phi_{\beta }{}_{\_\infty }$ = $\phi_{\alpha \_0}$) in neat PVDF at large strains. Since no quantitative information was found for the α → β phase transition kinetics, the phase transition constants were calibrated with the help of the overall material response extracted from the work of Defebvin [9]. The phase transition constants are listed in Table 2. The same model inputs were employed by Yan et al. [16] to capture the different stages of the mechanical behavior upon large-strain plastic deformation. As a consequence of the micromechanics homogenization concepts, it is worth noting that a key assumption is the phase transition parameters of the PVDF matrix in the composite are the same as those of the neat polymer. The identified parameters for the neat polymer are employed as direct inputs into the model to predict the composite response.

**Figure 2:**
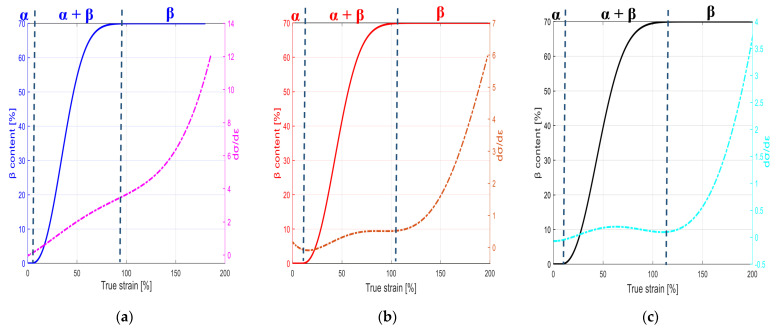
Evolution of hardening rate (right) and α → β phase transition (left) for neat PVDF as a function of strain for different temperatures: (**a**) 70 °C, (**b**) 90 °C and (**c**) 110 °C.

The temperature-dependent material response is plotted in the right scale of Figure 2 in the form of hardening rate. The response can be correlated with the sigmoid-type evolution of the newly formed β phase in the course of straining plotted in the left scale of the same figure. The incubation strain, from which α → β phase transition begins, and the strain level, from which full transformation is reached, are indicated by two vertical dashed lines. The former begins with the necking onset and the latter begins with the end of the necking propagation. The micro-mechanism of phase transition becomes less efficient when the temperature increases, and both strains are thus shifted with the temperature increase.

### 3.2. Piezoelectric Properties

**Figure 3:**
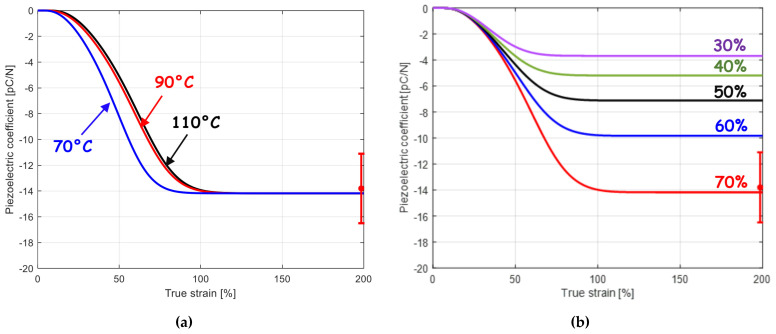
Piezoelectric coefficient evolution for neat PVDF as a function of strain (**a**) for different temperatures and (**b**) for different maximum phase transformation extents (lines: model, symbol: experimental data from [9]).

Figure 3 presents the numerical simulations of the neat PVDF piezoelectric coefficient in the course of straining. The figure shows the correlation of large-strain mesostructural properties and piezoelectric activity evolution. Figure 3a shows that the temperature influence on the micro-mechanism of α → β phase transition impacts the strain-induced piezoelectric coefficient evolution. The piezoelectric coefficient increases gradually with the α → β phase transition following a sigmoid-type evolution from its initial value $d_{33\_PVDF}^{0}$ for the non-polar crystalline α-phase (which does not exhibit inherent piezoelectricity) towards its stabilization corresponding to the final experimental value $d_{33\_PVDF}^{\infty }$ of the order of −14 ρC/N for β-PVDF [9,52,53]. Since a partial transition occurs between both, an obvious temperature dependence is observed. The gradual rate decrease in the piezoelectric coefficient as the applied strain increases and its stabilization value are closely related to the transformation micro-mechanism, as illustrated in Figure 3b by the obvious influence of the maximum occurrence of the β-phase.

**Figure 4:**
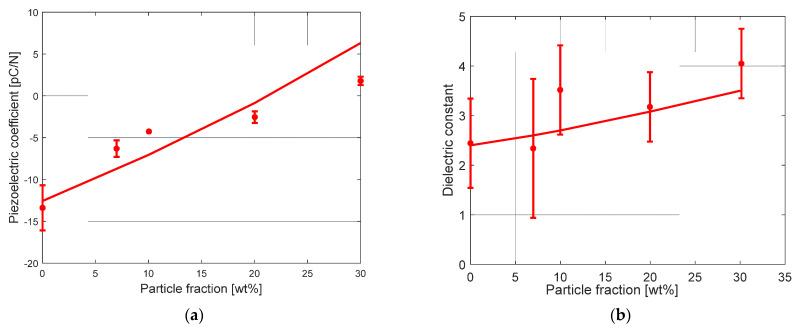
Piezoelectric coefficient (**a**) and dielectric constant (**b**) for BaTiO_3_–PVDF system as a function of BaTiO_3_ content (lines: model, symbols: experimental data from [9]).

Both the piezoelectric coefficient and the dielectric constant monotonically change with the BaTiO_3_ particle content. Indeed, because the ceramic piezoelectric coefficient counterbalances the PVDF piezoelectric coefficient (positive for the BaTiO_3_ particles and negative for the PVDF matrix), the piezoelectric effect of the polymer is progressively compensated by that of the ceramics. Figure 4 shows that the considerable role of the insertion of BaTiO_3_ particles on the piezoelectric properties is well captured by the model. Recall that the PVDF matrix is in its polar phase since the composite was previously strained. The neat β-PVDF showed a negative piezoelectric coefficient of the order of −14 pC/N, whereas the insertion of 30 wt% of particles into β-PVDF leads to a positive piezoelectric coefficient of about 1.5 pC/N, quite overestimated by the model.

**Figure 5:**
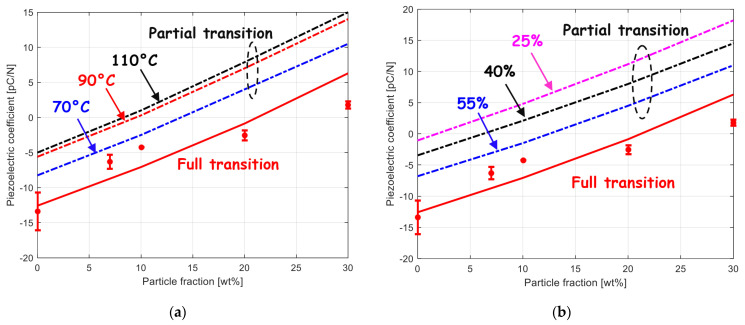
Piezoelectric coefficient for BaTiO_3_–PVDF system as a function of BaTiO_3_ content after transformation (**a**) at a strain level of 50% and different temperatures and (**b**) at a temperature of 90 °C and different strain levels (lines: model, symbols: experimental data from [9]).

According to the model estimates, the complete compensation of the two piezoelectric effects occurs at a filler content slightly higher than 20 wt%. The incorporation of the BaTiO_3_ particles leads to a decrease in the composite piezoelectric coefficient. Higher positive piezoelectric coefficient values could be obtained at higher filler concentrations. Nonetheless, from a certain filler concentration (about 50 wt%) the PVDF matrix cannot be in its polar phase form since the strain-induced phase transition cannot occur anymore due to a drastic loss of ductility [9,44]. As a relevant feature monitored by the model, it is possible to examine the effect of a partial transition on the piezoelectric coefficient. An illustrative example is presented in Figure 5a,b showing the effect of the variation of temperature (at a given applied strain) and the effect of the variation of applied strain (at a given temperature), respectively, both variations being related to a variation in β crystal extent. The composite piezoelectric behavior is modulated by the respective proportion of electroactive strained PVDF (negative coefficient) and piezoelectric particles (positive coefficient) with opposite signs of piezoelectric coefficient. The figure better highlights the role of the piezoelectric particle content.

### 3.3. Mechanical Response

**Figure 6:**
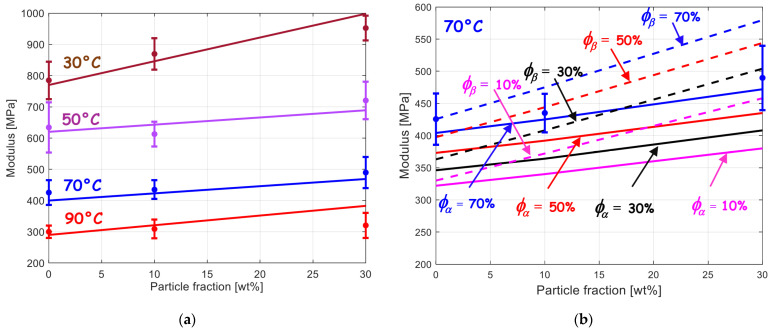
Elastic stiffness for BaTiO_3_–PVDF system as a function of BaTiO_3_ content: (**a**) with only α crystals at different temperatures and (**b**) with different amounts of α crystals or β crystals at the temperature of 70 °C (lines: model, symbols: experimental data from [9]).

The model simulations are compared to the experimental data in terms of composite stiffness in Figure 6a. It can be observed that the particle reinforcement is satisfactorily predicted by the micromechanical model over the range of particle concentrations for all the range of temperatures. The PVDF-based composites contain only α crystals. The phase transition effect on the overall stiffness can be examined thanks to the model. The occurrence of newly formed β crystalline phase leads to an enhanced composite stiffening, as illustrated in the example provided in Figure 6b.

### 3.4. Interfacial Debonding

**Figure 7:**
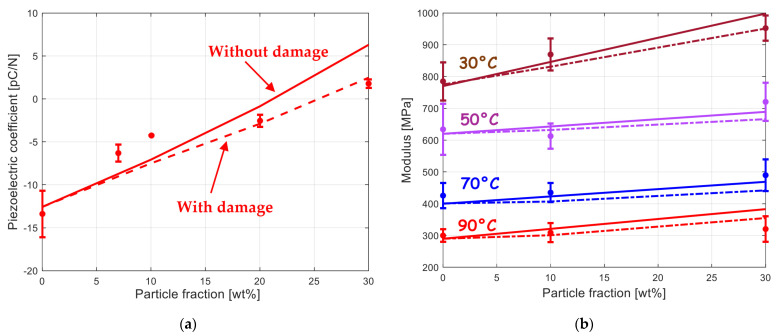
Role of interfacial damage on (**a**) piezoelectric coefficient as a function of BaTiO_3_ content and (**b**) elastic stiffness as a function of BaTiO_3_ content for different temperatures (solid lines: model without damage, dashed lines: model with damage, symbols: experimental data from [9]).

As a final point of discussion, the connection between the interfacial damage and the electroelastic properties are investigated numerically. Without having quantitative measurements of any damage indicator, it is proposed to calibrate the two damage parameters m and s on the piezoelectric coefficient and to further verify the model capacities on the mechanical response. The values are given in Table 2. As shown in Figure 7a and as expected, the experimental piezoelectric coefficient values are nearly perfectly caught by the model when the interfacial damage is considered. In this condition, the null piezoelectric response is estimated at a filler amount of 25 wt%. Whereas its effect is obvious on the piezoelectric coefficient, Figure 7b shows that the interfacial damage influences the stiffening more slightly, especially at the lowest particle concentrations.

**Figure 8:**
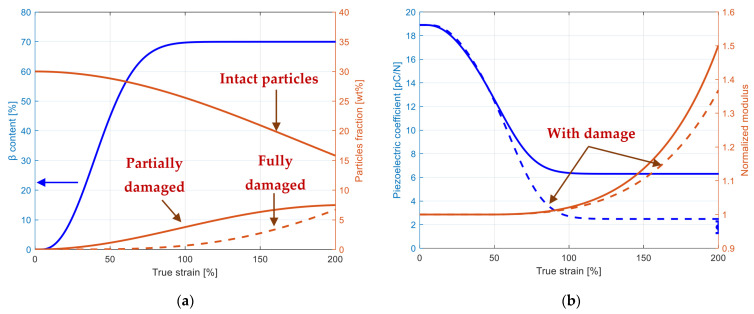
Evolution of mesostructural and electro-mechanical properties in BaTiO_3_–PVDF system with 30% particles: (**a**) partially/fully damaged particles and remaining intact particles (right scale) along with α → β phase transition (left scale) as a function of strain, (**b**) stiffness (right scale) and piezoelectric coefficient (left scale) as a function of strain.

By considering interfacial damage in the model, it becomes possible to capture more accurately the actual composite behavior and especially the increased electrical resistance. Figure 8a illustrates the progressive changes of the mesostructural properties in the course of straining for the particular example of the BaTiO_3_–PVDF system reinforced with 30% particles. The model can provide valuable insights on the microstructure–property relationship of PVDF-based piezo-composites and can help explain the experimental observations more effectively. More specifically, piezoelasticity changes due to the appearance of β crystals are clearly affected by the occurrence of interfacial debonding. The prejudice or the benefit of mesostructure changes on the overall response of the BaTiO_3_–PVDF composite is presented in Figure 8b. Under applied macroscopic deformation, the progressive degradation of interfaces acts as a weakening factor for both the piezoelectric coefficient and stiffness modulus. The phase transition stiffens the composite, but acts as a supplementary weakening factor for piezoelectric performances. A complex coupling exists between the micro-mechanisms of damage and of phase transition. The near-field direct interactions between the added BaTiO_3_ particles and the transformed PVDF matrix (replacing progressively the α inclusions by the β inclusions) are themselves affected by reason of changes in local properties.

## 4. Concluding Remarks

In this paper, a coupled electro-mechanical continuum-based model for semi-crystalline PVDF-based composites is presented to provide the necessary framework for the analysis of the relationship between mesostructural properties and electro-mechanical properties. In addition to the effective contributions of the inorganic particles in the composite response, the strain-induced α → β phase transition and the interfacial debonding between the PVDF matrix and particles are two deformation micro-mechanisms introduced into the model. A quantitative evaluation of the model was carried out on a BaTiO_3_–PVDF system initially crystallized in the non-polar crystalline α-phase and containing various concentrations of BaTiO_3_ particles. The model was used to clarify the influence of the occurrence of the β phase and microstructural damage in the course of straining on the electro-mechanical properties.

The model provides a useful tool to predict the electroelastic response of piezo-composites and could be applied to a wide range of filler types, such as whiskers, nanowires, or nanofibers, allowing better piezoelectric performances compared to spherical particles. The generality endowed by the electro-mechanical constitutive theory makes this application possible provided that, in addition to the filler content, the filler shape effect is explicitly considered in the piezoelectric activity. This necessary step deserves further work.

## Figures and Tables

**Table 1:** Elastic constants of α crystals, β crystals and BaTiO_3_ particles (values in GPa).

	$C_{11}$	$C_{22}$	$C_{33}$	$C_{44}$	$C_{55}$	$C_{66}$	$C_{12}$	$C_{13}$	$C_{23}$
α-PVDF [48]	16.10	15.70	148.1	0.5	3.9	6.5	6.2	8	0.8
β-PVDF [48]	19.83	24.50	287.33	1.17	0.33	1.67	4.33	0.83	3.33
BaTiO_3_ particles [21]	166	166	162	43	43	43	77	78	78

**Table 2:** Model constants.

	Parameter	Significance	Value
Amorphous Phase	$E_{g}$	Glassy modulus	3500 MPa
	$E_{r}$	Rubbery modulus	1500 MPa
	$\theta_{g}$	Glass transition temperature	−40.6 °C
	Δθ	Glass transition interval	20 °C
	$X_{g}$	Slope outside glass transition	−10 MPa/°C
	$\nu_{am}$	Rubbery Poisson’s ratio	0.4
Phase transition	${\dot{\varepsilon }}_{ref}$	Reference strain-rate	0.001/s
	$\alpha_{av}$	Avrami exponent	2.2
	Nu	Number density of nuclei	3.88 × 10^9^ (cm^−3^)
Interfacial debonding	m	Weibull parameter	2
	s	Weibull parameter	25

## Data Availability

The data presented in this study are available on request from the corresponding author.
